# Body Mass Index in Children Before, During, and After the COVID-19 Pandemic

**DOI:** 10.1001/jamanetworkopen.2025.19528

**Published:** 2025-07-09

**Authors:** Frederik Kirkemann Jensen, Sigrid Bjerge Gribsholt, Sara Schwartz, Anton Lund Andersen, Jens Meldgaard Bruun

**Affiliations:** 1 Steno Diabetes Centre Aarhus, Aarhus University Hospital, Aarhus, Denmark; 2 Danish National Centre for Obesity, Aarhus, Denmark; 3 Department of Endocrinology and Internal Medicine, Aarhus University Hospital, Aarhus, Denmark; 4 Department of Clinical Medicine, Aarhus University, Aarhus, Denmark

## Abstract

**Importance:**

Significant changes in body mass index (BMI; calculated as weight in kilograms divided by height in meters squared) have been observed in children during the COVID-19-pandemic; however, changes in different BMI categories after COVID-19 remain largely unknown.

**Objective:**

To examine changes in prevalence of underweight, overweight, and obesity in first- and sixth-grade children in Denmark during and after the COVID-19 pandemic compared with before COVID-19.

**Design, Setting, and Participants:**

This cross-sectional prevalence study was conducted from March 11, 2019, to January 31, 2024, using nationwide, population-based data from Danish health care registries. All children in first or sixth grade in Denmark during the COVID-19 time periods (before, during, and after) were eligible for inclusion. Children with outcome data (ie, anthropometric assessments) available in the Children’s Database were included in the analysis. Data were analyzed in March 2024.

**Exposure:**

National COVID-19 lockdowns.

**Main Outcomes and Measures:**

The outcome of interest was changes in age- and sex-adjusted BMI (iso-BMI). Using log-binomial regression, crude and adjusted prevalence ratios (aPRs) of iso-BMI categories (ie, underweight [<18.5], normal weight [18.5-24.9], overweight [25.0-29.9], and obesity [≥30.0]) were calculated using pre–COVID-19 levels as the reference, adjusting for sex, household income, and parental education.

**Results:**

A total of 268 761 first-grade children (137 826 [51.3%] male; 42 464 children [15.8%] with high household income; 172 678 children [64.3%] with parents with tertiary education) and 158 174 sixth-grade children (80 958 [51.2%] male; 34 798 children [22.0%] with high household income; 95 492 children [60.4%] with parents with tertiary education at baseline) were included. In first-grade children, a decrease in underweight was observed during COVID-19 (aPR, 0.76 [95% CI, 0.71-0.83]); contrarily, an increased prevalence of underweight was found in sixth-grade children after COVID-19 (aPR, 1.15 [95% CI, 1.06-1.24]). The prevalence of overweight increased in first- and sixth-grade children during COVID-19, but both returned to pre–COVID-19 levels afterwards (first grade: aPR, 0.97 [95% CI, 0.94-0.99]; sixth grade: aPR, 0.95 [95% CI, 0.93-0.98]). Likewise, the prevalence of obesity increased in first- and sixth-grade children during COVID-19 and remained elevated in first-grade children after COVID-19 (aPR, 1.12 [95% CI, 1.07-1.18]).

**Conclusions and Relevance:**

In this cross-sectional study of changes in iso-BMI in children in Denmark, prevalence of overweight and obesity increased during COVID-19. The prevalence of overweight declined after COVID-19, but the prevalence of obesity remained increased among first-grade children. Additionally, the prevalence of underweight increased in sixth-grade children after COVID-19. These findings highlight differences among children of varying ages and BMI classes.

## Key Points

**Question:**

Did prevalence of underweight, overweight, and obesity in schoolchildren change during and after the COVID-19 pandemic compared with before the pandemic?

**Findings:**

This cross-sectional study including 426 935 children in Denmark found a higher prevalence of obesity among children in first grade and underweight among children in sixth grade after the COVID-19 pandemic compared with before the pandemic.

**Meaning:**

This cross-sectional study found that body mass index outcomes of COVID-19 pandemic–related control policies and restrictions were not exclusively observed among children with obesity, which suggests that pandemic-related mitigation policies targeting children and adolescents in all body mass index categories are warranted.

## Teaser

This cross-sectional study examines changes in prevalence of underweight, overweight, and obesity in first- and sixth-grade children in Denmark during and after the COVID-19 pandemic compared with before COVID-19.

## Introduction

Adverse psychosocial and physical health effects exist in both ends of the body mass index (BMI; calculated as weight in kilograms divided by height in meters squared) spectrum. The number of children living with overweight or obesity is increasing worldwide,^1,2,3^ challenging mental well-being as well as increasing the risk of prematurely developing adverse health complications, such as prediabetes, diabetes,^4,5^ dyslipidemia,^6^ and metabolic-associated fatty liver disease.^7^ Likewise, underweight is associated with adverse psychosocial health and well-being and physical health problems, eg, stunted growth, weakened immune system, and impaired cognitive and academic performance.^8,9^ In Denmark, the prevalence of childhood underweight and obesity has remained relatively stable over the recent 2 decades.^9,10^

During the COVID-19 pandemic, Denmark entered a nationwide lockdown on March 11, 2020, with several pandemic-related control policies and restrictions being implemented, until all national restrictions were lifted on February 1, 2022.^11^ Several of the policies implemented to reduce transmission of COVID-19 focused on social distancing, which especially affected children, including widespread suspension of regular school time, with teaching being moved to online formats, as well as lockdown of recreational facilities, including sporting activities.^11^

Although not all of the adverse consequences of COVID-19 in adults are known, several have been reported^12,13^; however, the impact of the restrictions during the pandemic on children’s mental and physical health is not yet completely understood.^14^ School settings are known to contribute to upholding routines, such as sleep patterns, structured mealtimes, and regular physical activity.^14,15^ Therefore, the implementation of home quarantine may have affected children’s health behaviors with disrupted sleep, decrease in physical activity, and increase in sedentary behavior (eg, screen time), all placing children at an increased risk for deleterious health outcomes, including weight gain.^14,16,17,18,19,20,21^ However, post–COVID-19 and long-term consequences for children in all BMI categories remain poorly investigated.^22^

Thus, the aim of this cross-sectional study was to examine different age- and sex-adjusted BMI categories (iso-BMI; classified as underweight, <18.5; normal weight, 18.5-24.9; overweight, 25.0-29.9; and obesity, ≥30.0) among Danish schoolchildren during and after, compared with before, the COVID-19 pandemic. We hypothesized that children exhibited substantial changes within each BMI category during COVID-19, reversing after COVID-19, when children returned to their daily routines. A secondary aim was to assess potential associations of children’s age, sex, and socioeconomic status with BMI.

## Methods

This cross-sectional study was registered with the Danish Data Protection Agency. According to Danish legislation, registry-based studies do not require separate approval from the Danish Research Ethics Committee or informed consent. The study was designed in accordance with the Strengthening the Reporting of Observational Studies in Epidemiology (STROBE) reporting guideline for cross-sectional studies.

### Study Design and Setting

We conducted a retrospective prevalence study using a repeated cross-sectional approach based on Danish registry data, from March 11, 2019, to January 31, 2024. We established 3 time periods (eFigure 1 in Supplement 1). The period before COVID-19 was defined as March 11, 2019, to March 10, 2020, and served as the baseline period. The second period, during COVID-19, spanned from September 1, 2020, to June 30, 2021. This start date was chosen to allow for anthropometric changes in the population to stabilize and eliminate any carryover effects. The third period, after COVID-19, started 6 months from the penultimate lift of restrictions, August 1, 2022, to January 31, 2024 (eFigure 1 in Supplement 1).

Denmark has an extensive welfare state, providing universal tax-supported health care for its citizens, and on birth or immigration, all residents are registered in the Civil Registration System with a unique personal identification number (CPR number) allowing exact data linkage across various database records.^23,24^ Using pseudoanonymized CPR numbers, we linked general personal data (date of birth, age, sex, and CPR number of parents) from the Civil Registration System with data from the Children’s Database^25^ and from the Integrated Database for Labor Market Research.^26^

Danish public school comprises of 10 years of tax-supported free-of-charge primary and lower secondary education for children starting at age 6 years, including obligatory health examinations with measurements of height and weight, registered in the Children’s Database.^25,27^ The Integrated Database for Labor Market Research is overseen by Statistics Denmark and stores data on the highest completed education and income of Danish residents.^26^

### Study Population

This study included all children who had available data on anthropometry (height and weight) at ages 7 and 13 years (corresponding to children in first and sixth grades, respectively) in the Children’s Database in any of the 3 study periods. Children aged ±9 months from either age (7 or 13 years) were included in the age groups to account for children enrolled early or late. As anthropometric measurements were conducted at specific grades, each child could only attribute 1 measurement in the analysis. In case multiple measurements were registered, only the most recent measurement was included.

### Primary Outcome Measure

The study’s primary outcome was changes in age- and sex-adjusted BMI (iso-BMI) categories, grouped according to World Health Organization standards: underweight (iso-BMI, <18.5), normal weight (iso-BMI, 18.5-24.9), overweight (iso-BMI, 25.0-29.9), and obesity (iso-BMI, ≥30.0).^28^ Exact cutoffs for iso-BMI categories were defined using the Extended International Body Mass Index Cut-Offs,^29^ which are standardized for sex and exact age in months for children between the ages of 2 and 18 years.

### Covariates

Covariates, including confounders, were identified based on a review of relevant literature.^1,3,4,14,16,17,30,31,32,33,34^ Other than age (7 and 13 years), the sex (boy or girl) and socioeconomic status of the children were included in the analysis and adjusted for as possibly confounding variables. Socioeconomic status was accounted for by using variables for parental income and education level as a proxy from the Integrated Database for Labor Marked Research. Data on parents’ equivalized disposable household income was used, allowing us to account for separated parents and alternative family structures, while also adjusting for potential economies of scale in different family sizes (hereafter, referred to as *household income*). Household income was categorized using quartiles for yearly gross equivalized disposable income in Denmark as cutoffs (low, <25%; lower-middle, 25%-49.9%; upper-middle, 50%-74.9%; and high, ≥75%). Likewise, data on parents’ highest completed education were grouped as primary (primary and lower secondary education), secondary (upper secondary education), and tertiary (short-cycle tertiary, Bachelor’s, or higher education) according to International Standard Classification of Education Standards.^35^ After being grouped, either parent with the highest level of income and education, respectively, was included in the analysis.

### Statistical Analysis

Baseline characteristics and distribution of covariates in the study population were cross-tabulated by the 3 pandemic time periods, stratified by school grade. Prevalence and prevalence differences in iso-BMI categories in the 3 time periods were calculated, stratified by school grade. In addition, annual crude prevalences of iso-BMI categories were computed from 2011 to 2019 (pre-pandemic) and for each BMI category, and an informal interrupted time-series (ITS) model was fitted for the periods during and after COVID-19, stratified by school grade. The observed prevalences of iso-BMI categories during and after the pandemic were then plotted against the estimation models to assess conformity with pre–COVID-19 trends. Characteristics of the final study population were cross-tabulated with the base population of eligible Danish children, as well as excluded children, to assess the representativity and robustness of the data.

We fitted separate models for children in first and sixth grades, by which crude prevalence ratios (PRs) and adjusted PRs (aPRs) were computed for each binary iso-BMI category. Log-binomial regression was used to model PRs.^36^ In cases where the log-binomial model failed to converge, we used modified Poisson regression to compute the PRs and aPRs.^37^ For all models, aPRs were adjusted for sex, household income, and parental education. All statistical analyses were carried out using Stata/BE version 18.0 for Windows (StataCorp). *P* values were 2-sided, and statistical significance was set at *P* ≤ .05. Data were analyzed in March 2024.

## Results

Of the 457 114 Danish first-grade children and 503 552 sixth-grade children eligible for inclusion in the 3 time periods, a total of 268 761 first-grade children (137 826 [51.3%] male; 42 464 children [15.8%] with high household income; 172 678 children [64.3%] with parents with tertiary education) and 158 174 sixth-grade children (80 958 [51.2%] male; 34 798 children [22.0%] with high household income; 95 492 children [60.4%] with parents with tertiary education at baseline) had outcome data available and were included in the analysis (eFigure 2 in Supplement 1). Across all 3 time periods, more children were boys (50.1%-51.9%), lived in upper-middle class households (40.4%-42.7%), and had parents with tertiary education levels (58.2%-66.2%) (Table).

**Table.** Baseline Sociodemographic Characteristics of the Study Population

Characteristic	Children by COVID-19 period, No (%)^a^			
	Before	During	After	Overall
**First-grade children**				
Total	70 588 (26.3)	78 411 (29.2)	119 762 (44.6)	268 761 (100)
Body mass index group^b^				
Underweight	1397 (2.0)	1197 (1.5)	2518 (2.1)	5112 (1.9)
Normal weight	57 985 (82.2)	62 238 (79.4)	98 534 (82.3)	218 757 (81.4)
Overweight	8563 (12.1)	10 952 (14.0)	13 840 (11.6)	33 355 (12.4)
Obesity	2643 (3.7)	4024 (5.1)	4870 (4.1)	11 537 (4.3)
Sex				
Male	35 839 (50.8)	40 269 (51.4)	61 718 (51.5)	137 826 (51.3)
Female	34 749 (49.2)	38 142 (48.6)	58 044 (48.5)	130 935 (48.7)
Household income^c^				
Low	3717 (5.3)	4049 (5.2)	5397 (4.5)	13 163 (4.9)
Lower-middle	25 331 (35.9)	29 649 (37.8)	45 171 (37.7)	100 151 (37.3)
Upper-middle	29 418 (41.7)	31 678 (40.4)	48 949 (40.9)	110 045 (41.0)
High	11 740 (16.6)	12 562 (16.0)	18 162 (15.2)	42 464 (15.8)
Missing	382 (0.5)	473 (0.6)	2083 (1.7)	2938 (1.1)
Parental education^d^				
Primary	4453 (6.3)	4824 (6.2)	6184 (5.2)	15 461 (5.8)
Secondary	21 078 (29.9)	22 328 (28.5)	32 206 (26.9)	75 612 (28.1)
Tertiary	43 494 (61.6)	49 955 (63.7)	79 229 (66.2)	172 678 (64.3)
Missing	1563 (2.2)	1304 (1.7)	2143 (1.8)	5012 (1.9)
**Sixth-grade children**				
Total	51 773 (32.7)	31 014 (19.6)	75 387 (47.7)	158 174 (100)
Body mass index group^b^				
Underweight	1101 (2.1)	635 (2.1)	1837 (2.4)	3573 (2.3)
Normal weight	39 829 (76.9)	23 053 (74.3)	58 369 (77.4)	121 251 (76.7)
Overweight	8550 (16.5)	5614 (18.1)	11 799 (15.7)	25 963 (16.4)
Obesity	2293 (4.4)	1712 (5.5)	3382 (4.5)	7387 (4.7)
Sex				
Male	26 308 (50.8)	15 550 (50.1)	39 100 (51.9)	80 958 (51.2)
Female	25 465 (49.2)	15 464 (49.9)	36 287 (48.1)	77 216 (48.8)
Household income^c^				
Low	2103 (4.1)	1239 (4.0)	2663 (3.5)	6005 (3.8)
Lower-middle	15 574 (30.1)	9849 (31.8)	23 231 (30.8)	48 654 (30.8)
Upper-middle	21 867 (42.2)	13 246 (42.7)	32 041 (42.5)	67 154 (42.5)
High	12 026 (23.2)	6542 (21.1)	16 230 (21.5)	34 798 (22.0)
Missing	203 (0.4)	138 (0.4)	1222 (1.6)	1563 (1.0)
Parental education^d^				
Primary	3213 (6.2)	1883 (6.1)	3963 (5.3)	9059 (5.7)
Secondary	17 541 (33.9)	10 348 (33.4)	23 020 (30.5)	50 909 (32.2)
Tertiary	30 125 (58.2)	18 327 (59.1)	47 040 (62.4)	95 492 (60.4)
Missing	894 (1.7)	456 (1.5)	1364 (1.8)	2714 (1.7)

^a^ COVID-19 time periods were defined as before, March 11, 2019, to March 10, 2020; during, September 1, 2020, to June 30, 2021; and after, August 1, 2022, to January 31, 2024.

^b^ Age- and sex-adjusted body mass index (calculated as weight in kilograms divided by height in meters squared) categories from Extended International Body Mass Index Cut-Offs: underweight, less than 18.5; normal weight, 18.5 to 24.9; overweight, 25.0 to 29.9; and obesity, 30 or greater.

^c^ Parental equivalized disposable household income categorized by Danish year-specific quartiles, low, less than 25%; lower-middle, 25% to 49.9%; upper-middle, 50% to 74.9%; and high, 75% or greater.

^d^ International Standard Classification of Education level of parents’ highest completed education.

In the 1-year time period before COVID-19, prevalence of underweight was similar in both grades, at 2.0% (95% CI, 1.9%-2.1%) among first-grade children and 2.1% (95% CI, 2.0%-2.3%) among sixth-grade children. The prevalence of overweight was 12.1% (95% CI, 11.9%-12.4%) among first-grade children and 16.5% (95% CI, 16.2%-16.8%) among sixth-grade children, and the prevalence of obesity was 3.7% (95% CI, 3.6%-3.9%) among first-grade children and 4.4% (95% CI, 4.3%-4.6%) among sixth-grade children (eTable 1 in Supplement 1). Tabulated characteristics showed comparable distributions of covariates between those with and without anthropometric data (eTable 2 in Supplement 1), although excluded children were slightly more likely to have higher iso-BMI and lower household income (eTable 3 in Supplement 1).

**Figure 1.**
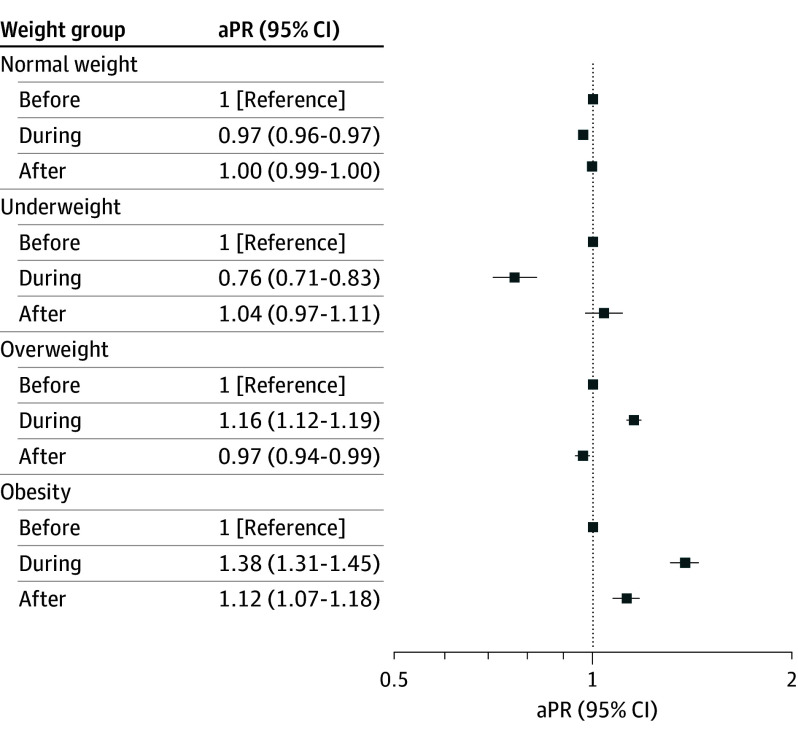
Adjusted Prevalence Ratios (aPRs) of Age- and Sex-Adjusted Body Mass Index (BMI) Categories in First-Grade Children in Denmark by COVID-19 Period BMI was calculated as weight in kilograms divided by height in meters squared and adjusted for sex of the child, parental education, and household income. Underweight was defined as BMI less than 18.5; normal weight, 18.5 to 24.9; overweight, 25.0 to 29.9; and obesity, 30.0 or greater.

**Figure 2.**
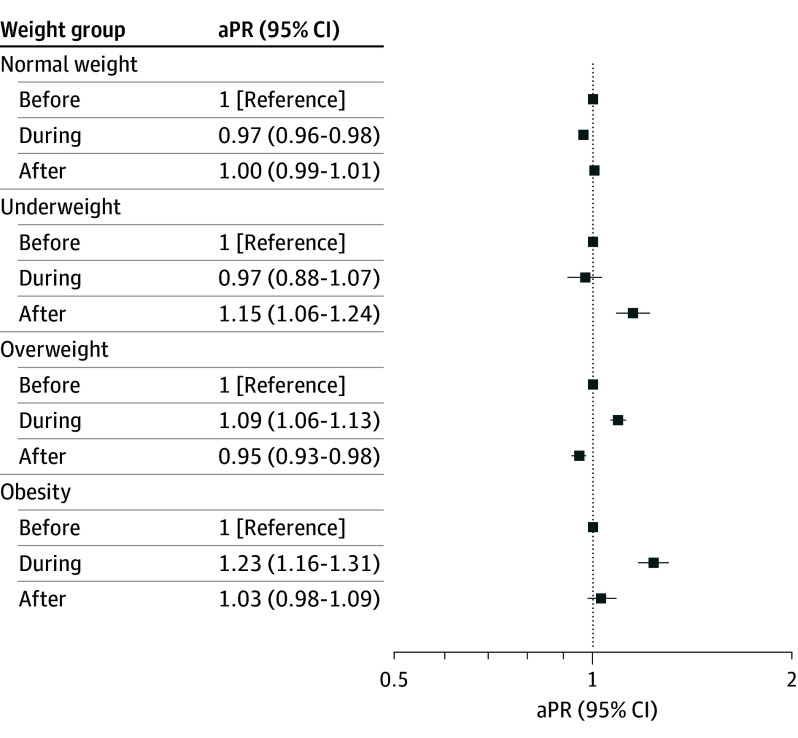
Adjusted Prevalence Ratios (aPRs) of Age- and Sex-Adjusted Body Mass Index (BMI) Categories in Sixth-Grade Children in Denmark by COVID-19 Period BMI was calculated as weight in kilograms divided by height in meters squared and adjusted for sex of the child, parental education, and household income. Underweight was defined as BMI less than 18.5; normal weight, 18.5 to 24.9; overweight, 25.0 to 29.9; and obesity, 30.0 or greater.

Compared with pre–COVID-19 levels, underweight decreased during COVID-19 among first-grade children (aPR, 0.76 [95% CI, 0.71-0.83]) but remained unchanged among sixth-grade children (aPR, 0.97 [95% CI, 0.88-1.07]). During the post–COVID-19 period, underweight levels returned to prepandemic levels for first-grade children (aPR, 1.04 [95% CI, 0.97-1.11]) (Figure 1); however, underweight increased significantly for sixth-grade children (aPR, 1.15 [95% CI, 1.06-1.24]) (Figure 2). Prevalence of overweight increased during COVID-19 in children in first grade (aPR, 1.16 [95% CI, 1.12-1.19]) and sixth grade (aPR, 1.09 [95% CI, 1.06-1.13]) and decreased during the post–COVID-19 period (first grade: aPR, 0.97 [95% CI, 0.94-0.99]; sixth grade: aPR, 0.95 [95% CI, 0.93-0.98]) (Figure 1 and Figure 2). Likewise, obesity increased during COVID-19 (first grade: aPR, 1.38 [95% CI, 1.31-1.45]; sixth grade: aPR, 1.23 [95% CI, 1.16-1.31]). Post–COVID-19 obesity prevalence decreased in children in both grades (Figure 1 and Figure 2); however, obesity prevalence remained elevated among first grade children (aPR, 1.12 [95% CI, 1.07-1.18]).

For all time periods, higher household income and parental education were associated with lower prevalence of overweight and obesity. Higher parental education was associated with an increased prevalence of underweight, while higher household income was associated with decreased prevalence. Among children in first grade, girls exhibited higher prevalences of overweight and obesity and lower prevalence of underweight compared with boys; this pattern was reversed among children in sixth grade, where boys showed higher prevalences of overweight and obesity and lower prevalence of underweight.

**Figure 3.**
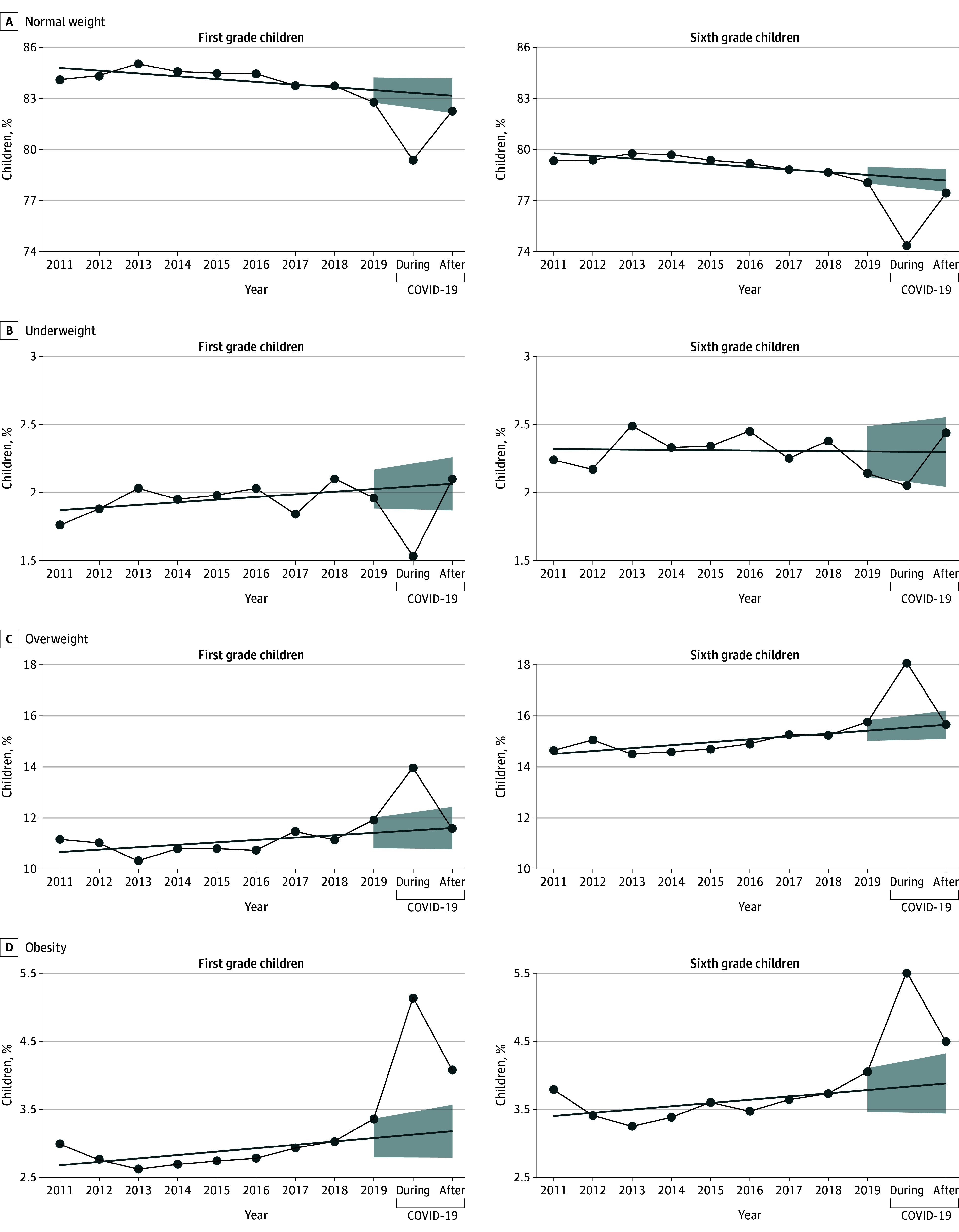
Annual Prevalence of Age- and Sex-Adjusted Body Mass Index (BMI) Categories in First and Sixth Grade Children in Denmark and Linear Projection Models for the COVID-19 Period BMI was calculated as weight in kilograms divided by height in meters squared and adjusted for sex of the child, parental education, and household income. Underweight was defined as BMI less than 18.5; normal weight, 18.5 to 24.9; overweight, 25.0 to 29.9; and obesity, 30.0 or greater. Projection models were calculated from prepandemic trends; shaded areas indicate 95% CIs of the projections.

Figure 3 illustrates an ITS model of crude iso-BMI prevalence before COVID-19 and computed prevalences during and after COVID-19. During COVID-19, prevalences of underweight, overweight, and obesity all diverged significantly from the model’s projections. In the post–COVID-19 period, underweight and overweight trends returned within the model’s 95% CI, while obesity remained elevated in children in both grades.

## Discussion

To our knowledge, this large cross-sectional prevalence study is the first study to report on differences in prevalence of iso-BMI groups in Danish children in first and sixth grades during and after the COVID-19 pandemic compared with before COVID-19. In both grades, significant increases in overweight and obesity were observed during COVID-19. In the post–COVID-19 period, prevalence of overweight was comparable to pre–COVID-19 levels; however, prevalence of obesity remained elevated by 12.4% in first-grade children. Prevalence of underweight was different for children in the 2 grades, with a decrease during COVID-19 for children in first grade, returning to pre–COVID-19 levels after. For children in sixth grade, the prevalence of underweight during COVID-19 was comparable to pre–COVID-19 levels but increased significantly after COVID-19, at approximately 14.7% higher compared with pre–COVID-19 levels.

Globally, an increase in children living with overweight and obesity has occurred over recent decades,^1,3,33^ and any observed increase in our study may reflect these pre-existing tendencies in society rather than COVID-19–specific effects. However, the prevalences of all iso-BMI categories diverged from linear estimations during COVID-19, suggesting an effect of the pandemic lockdown on pediatric BMI distribution. After COVID-19, most iso-BMI categories returned to the projection line, indicating a temporary effect of the lockdowns and control policies. However, first-grade children did exhibit continuously higher aPRs of obesity after COVID-19, and children in first and sixth grades had crude obesity prevalences that were higher than the ITS projection model would suggest. This may indicate that while the post–COVID-19 aPR of obesity in sixth-grade children showed no significant difference from the prevalence in the year preceding COVID-19, both age groups had higher-than-expected obesity prevalences based on trends from 2011 to 2019. Contrarily, while children in sixth grade exhibited an increased aPR of underweight after COVID-19, the prevalence did not differ significantly from long-term trends since 2011. However, these are crude estimates of prevalence, as opposed to the computed prevalence ratios that are adjusted for age and socioeconomic status.

Our findings of increased childhood overweight and obesity during the COVID-19 pandemic are largely in agreement with previous studies from other countries.^14,16,19,32^ However, only few international studies have examined overweight and obesity trends in children after COVID-19, and these have used either self-reported questionnaire data^21^ or small population sizes.^22,38^ One study from Sweden examined 50 833 preschool children aged 3 to 5 years and found increases in overweight and obesity during COVID-19 for children aged 3 and 4 years, decreasing again in the late– and post–COVID-19 periods, while children aged 5 years exhibited no change in BMI.^39^ To our knowledge, no prior studies have examined post–COVID-19 prevalence of underweight, overweight, and obesity in children and adolescents, extending as late as 2024, although some studies have reported plateaus or decreases in overweight and obesity toward the end of the COVID-19 pandemic.^38,40^ In line with our findings, another study reported a decrease in pediatric overweight but a continued increase in obesity,^21^ highlighting possible differences in how overweight and obesity affect children.

Internationally, the impact of COVID-19 varied across countries, resulting in different strategies that may complicate comparisons among countries. While this study identified 3 periods related to the COVID-19 pandemic in Denmark, other studies on pediatric BMI during COVID-19 have used alternate cutoff dates for their measurement periods.^21,38,39^ Of these, some have suggested that changes in pediatric BMI due to external effects may present themselves very quickly, sometimes in as soon as 3 weeks.^20,41^ For our study, we decided to include a 6-month wash out/weight stabilizing period from the initial lockdown to eliminate any carry-over effects and capture more persisting weight changes.

Our results indicate that the cross-sectional prevalence in iso-BMI categories varied by age, with first-grade children exhibiting greater increases in prevalence of overweight and obesity, with obesity remaining elevated after COVID-19. Conversely, underweight was attenuated among first-grade children during COVID-19 but augmented among sixth-grade children after COVID-19. These findings suggest that the 2 grade groups are not directly comparable, possibly due to physiological changes related to hormonal changes in adolescence, eg, with children in sixth grade experiencing the effects of puberty,^42^ highlighting the need for differentiated age-specific approaches. Other studies point to similar tendencies, with some reporting larger prevalence changes for younger age groups^20,43^; however, some studies have also observed higher increases in prevalence among sixth-grade children.^44^ This may imply that beyond biological factors, such as age and sex, exact changes in iso-BMI may be influenced by economic and cultural differences, with our results indicating that in a Danish context, younger children are more susceptible to adverse weight-related effects from sudden changes in their daily routines, as seen during the COVID-19 lockdowns. No firm conclusions can be drawn from this study alone, as the study design is cross-sectional and descriptive by nature and does not permit causal interpretations. Further research, including results from other countries, is therefore warranted.

### Limitations

This study has some limitations. Using weight and height data collected in routine school health examinations may not be as accurate as data collected for research purposes. Any misclassification of the outcome would be assumed to be nondifferential but could lead to increased variance and an underestimation of the study’s findings.

Using anthropometric measurements from routine school health examinations at predetermined school grades restricted the study to focus on children in first and sixth grades (ages 7 and 13 years) and did not permit for the same cohort of children being followed-up throughout the 3 COVID-19 time periods, which can be seen as a limitation for the study. Additionally, BMI trends in preschool children are highly relevant and may be a valuable focus in future research.

The final study sizes differed significantly between first-grade and sixth-grade children, with the younger group being larger. Routine health checkups performed in schools are required to be offered, but children and parents can refuse, which may explain the lower participation among sixth-grade children. Body weight is particularly sensitive information, and concerns about stigmatization might lead some children, particularly those with higher BMI categories, to opt out of measurements, potentially introducing selection bias. However, the included study population had similar distributions of covariates compared with the overall population, strengthening the generalizability of the computed iso-BMI distributions. More exclusions occurred among children with higher BMI categories and lower income groups, potentially leading to an underestimation of associations.

For the cross-sectional analysis, we computed PRs adjusted for sex of the child, household income, and parental education, in line with other studies examining trends in pediatric BMI.^16,20,40,41,45^ Nevertheless, residual confounding can occur; however, confounding is unlikely to create an association but instead skew estimates toward the crude, meaning the study findings may underestimate the true extent of our finding in the different BMI categories during the pandemic.

## Conclusions

In this cross-sectional study of changes in iso-BMI in children in Denmark, we found differences in overweight and obesity among first- and sixth-grade children during COVID-19, with first-grade children exhibiting a continued increase in obesity during the post–COVID-19 period. Conversely, prevalence of underweight was attenuated in first-grade children during COVID-19 but unchanged in sixth-grade children, and the latter group demonstrated an increase post–COVID-19. The more pronounced fluctuations in BMI observed in children in first grade may suggest age-related differences in how COVID-19–related changes in daily life impact BMI. This implies the need for age-specific care, especially for younger children, in extreme situations, like a pandemic. Whether these findings apply more broadly to younger children requires further studies.

## Notes

Supplement 1. **eFigure 1**. Timeline of COVID-19 pandemic period in Denmark and selection of study time periods for inclusion of anthropometric measurements **eFigure 2.** Flowchart for selection of study population **eTable 1.** Prevalence, absolute prevalence difference, and crude prevalence ratios of iso-BMI weight categories in Danish first- and sixth-grade children by COVID-19 time periods **eTable 2.** Distribution in sociodemographic covariates in children with and without anthropometric assessment available at first and sixth grade **eTable 3.** Distribution across outcome and sociodemographic covariates in 1st and 6th grade children excluded from the study population due to non-complete data availability

Supplement 2.
Data Sharing Statement
