# Supplementary material for: Body Mass Index in Children Before, During, and After the COVID-19 Pandemic
Source: JAMA Netw Open. 2025 Jul 9;8(7):e2519528. doi: 10.1001/jamanetworkopen.2025.19528 (PMC12242698; doi:10.1001/jamanetworkopen.2025.19528)
Supplement: Supplement 1. — eFigure 1. Timeline of COVID-19 pandemic period in Denmark and selection of study time periods for inclusion of anthropometric measurements eFigure 2. Flowchart for selection of study population eTable 1. Prevalence, absolute prevalence difference, and crude prevalence ratios of iso-BMI weight categories in Danish first- and sixth-grade children by COVID-19 time periods eTable 2. Distribution in sociodemographic covariates in children with and without anthropometric assessment available at first and sixth grade eTable 3. Distribution across outcome and sociodemographic covariates in 1st and 6th grade children excluded from the study population due to non-complete data availability [file jamanetwopen-e2519528-s001.pdf]

Supplemental Online Content

Jensen FK, Gribsholt SB, Schwartz S, Andersen AL, Bruun JM. Body mass index in children before, during, and after the COVID-19 pandemic. *JAMA Netw Open*. 2025;8(7):e2519528. doi:10.1001/jamanetworkopen.2025.19528:

eFigure 1. Timeline of COVID-19 pandemic period in Denmark and selection of study time periods for inclusion of anthropometric measurements
eFigure 2. Flowchart for selection of study population
eTable 1. Prevalence, absolute prevalence difference, and crude prevalence ratios of iso-BMI weight categories in Danish first- and sixth-grade children by COVID-19 time periods
eTable 2. Distribution in sociodemographic covariates in children with and without anthropometric assessment available at first and sixth grade
eTable 3. Distribution across outcome and sociodemographic covariates in 1st and 6th grade children excluded from the study population due to non-complete data availability

This supplemental material has been provided by the authors to give readers additional information about their work.

© 2025 Jensen FK et al. *JAMA Network Open*.

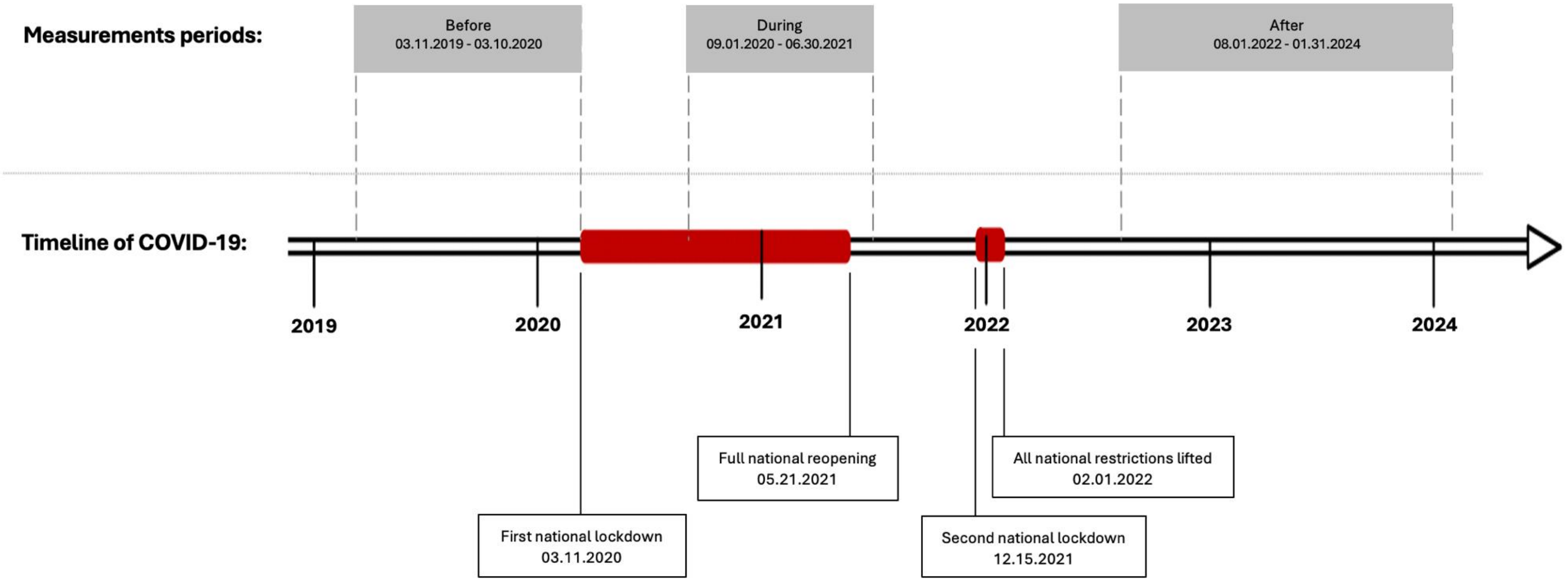

eFigure 1: Timeline of COVID-19 pandemic period in Denmark and selection of study time periods for inclusion of anthropometric measurements

© 2025 Jensen FK et al. *JAMA Network Open*.

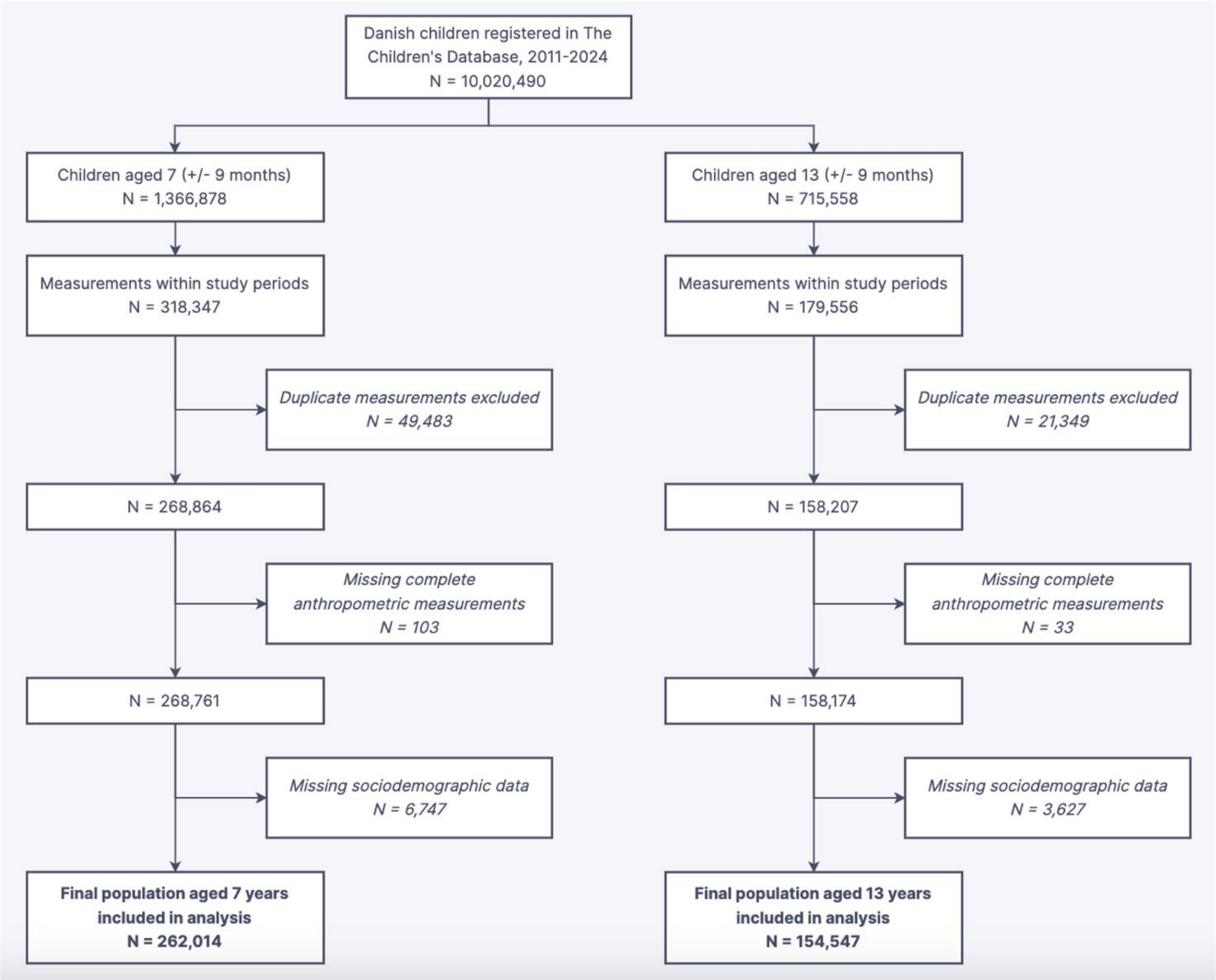

eFigure 2: Flowchart for selection of study population

© 2025 Jensen FK et al. *JAMA Network Open*.

eTable 1: Prevalence, absolute prevalence difference, and crude prevalence ratios of iso-BMI weight categories in Danish 1st and 6th grade children by COVID-19 time periods

	1st grade children			6th grade children		
**Iso-BMI weight group[a]**	**Prev. [95% CI][b]**	**Prev. diff.[c]**	**PR [95% CI][d]**	**Prev. [95% CI][b]**	**Prev. diff.[c]**	**PR [95% CI][d]**
**Normal Weight**						
Before	0.821 [0.819; 0.824]	-	1 [ref.]	0.769 [0.766; 0.773]	-	1 [ref.]
During	0.794 [0.791; 0.797]	− 0.027	0.97 [0.96; 0.97]	0.743 [0.738; 0.748]	− 0.026	0.97 [0.96; 0.97]
After	0.823 [0.821; 0.825]	+ 0.029	1.00 [1.00; 1.01]	0.774 [0.771; 0.777]	+ 0.031	1.01 [1.00; 1.01]
**Underweight**						
Before	0.020 [0.019; 0.021]	-	1 [ref.]	0.021 [0.020; 0.023]	-	1 [ref.]
During	0.015 [0.014; 0.016]	− 0.005	0.77 [0.72; 0.83]	0.020 [0.019; 0.022]	− 0.001	0.96 [0.87; 1.06]
After	0.021 [0.020; 0.022]	+ 0.006	1.06 [1.00; 1.13]	0.024 [0.023; 0.025]	+ 0.004	1.15 [1.06; 1.23]
**Overweight**						
Before	0.121 [0.119; 0.124]	-	1 [ref.]	0.165 [0.162; 0.168]	-	1 [ref.]
During	0.140 [0.137; 0.142]	+ 0.019	1.15 [1.12; 1.18]	0.181 [0.177; 0.185]	+ 0.016	1.10 [1.06; 1.13]
After	0.116 [0.114; 0.117]	− 0.024	0.95 [0.93; 0.98]	0.157 [0.154; 0.159]	− 0.024	0.95 [0.92; 0.97]
**Obesity**						
Before	0.037 [0.036; 0.039]	-	1 [ref.]	0.044 [0.043; 0.046]	-	1 [ref.]
During	0.051 [0.050; 0.053]	+ 0.014	1.37 [1.31; 1.44]	0.055 [0.053; 0.058]	+ 0.011	1.25 [1.17; 1.33]
After	0.041 [0.040; 0.042]	− 0.010	1.09 [1.04; 1.14]	0.045 [0.043; 0.046]	− 0.010	1.01 [0.96; 1.07]

[95% CI] = 95% confidence intervals. COVID-19 time periods; Before (11/3/19-10/3/20), During (1/9/20-30/6/ 21), After (1/8/22-31/1/24).

[a] Age- and sex-adjusted Body Mass Index (iso-BMI) weight categories from IOTF thresholds; underweight (iso-BMI ≤ 18.5 kg/m^2), normal weight (iso-BMI 18.5-24.9 kg/m^2), overweight (iso-BMI 25-30 kg/m^2), obesity (iso-BMI ≥ 30 kg/m^2). [b] Prevalence of iso-BMI weight group by pandemic period. [b] numeric prevalence difference from prior pandemic period. [c] Prevalence ratio as compared to pandemic period “Before” within each iso-BMI weight group.

© 2025 Jensen FK et al. *JAMA Network Open*.

eTable 2: Distribution in sociodemographic covariates in children with and without anthropometric assessment available at 1st and 6th grade

	Children with AE, No [%]	Children without AE, No [%]	Overall, No [%]
**1st grade children**			
**Sex**			
Male	137,826 [51.3]	96,614 [51.3]	234,440 [51.3]
Female	130,935 [48.7]	91,766 [48.7]	222,701 [48.7]
**Household Income[a]**			
Low	13,163 [4.9]	11,150 [5.9]	24,313 [5.3]
Lower-middle	100,151 [37.3]	69,346 [36.8]	169,497 [37.1]
Upper-middle	110,045 [41.0]	74,557 [39.6]	184,602 [40.4]
High	42,464 [15.8]	29,516 [15.7]	71,980 [15.8]
Missing	2,938 [1.1]	3,814 [2.0]	6,752 [1.5]
**Parental Education[b]**			
Primary	15,461 [5.8]	11,791 [6.3]	27,252 [6.0]
Secondary	75,612 [28.1]	51,878 [27.5]	127,490 [27.9]
Tertiary	172,678 [64.3]	120,539 [64.0]	293,217 [64.1]
Missing	5,010 [1.9]	4,175 [2.2]	9,185 [2.0]
**Total**	**268,761 [58.8]**	**188,383 [41.2]**	**457,114 [100]**
**6th grade children**			
**Sex**			
Male	80,958 [51.2]	176,915 [51.2]	257,873 [51.2]
Female	77,216 [48.8]	168,463 [48.8]	245,679 [48.8]
**Household Income[a]**			
Low	6,005 [3.8]	14,869 [4.3]	20,874 [4.2]
Lower-middle	48,654 [30.8]	109,146 [31.6]	157,800 [31.3]
Upper-middle	67,154 [42.5]	142,956 [41.4]	210,110 [41.7]
High	34,798 [22.0]	74,407 [21.5]	109,205 [21.7]
Missing	1,563 [1.0]	4,000 [1.2]	5,563 [1.1]
**Parental Education[b]**			
Primary	9,059 [5.7]	21,709 [6.3]	30,768 [6.1]
Secondary	50,909 [32.2]	108,560 [31.4]	159,469 [31.7]
Tertiary	95,492 [60.4]	208,741 [60.4]	304,233 [60.4]
Missing	2,714 [1.7]	6,368 [1.8]	9,082 [1.8]
**Total**	**158,174 [31.4]**	**345,378 [68.6]**	**503,552 [100]**

[a] Parental equivalized disposable household income categorized by Danish year-specific quartiles, low (<25%), lower-middle (25-49.9 %), upper-middle (50-75%), high (>75%). [b] International Standard Classification of Education (ISCED) level of parents' highest completed education.

© 2025 Jensen FK et al. *JAMA Network Open*.

eTable 3: Distribution across outcome and sociodemographic covariates in 1st and 6th grade children excluded from the study population due to non-complete data availability

	Study population, No [%]	Excluded, No [%]	Overall, No [%]
**1st grade children**			
**Weight Group[a]**			
Underweight	4,961 [1.89]	151 [2.24]	5,112 [1.90]
Normal weight	213,565 [81.51]	5,192 [76.95]	218,757 [81.39]
Overweight	32,411 [12.37]	944 [13.99]	33,355 [12.41]
Obesity	11,077 [4.23]	460 [6.82]	11,537 [4.30]
**COVID-19 pandemic period[b]**			
Before	69,022 [26.34]	1,641 [23.96]	70,663 [26.28]
During	76,778 [29.30]	1,649 [24.07]	78,427 [29.17]
After	116,214 [44.35]	3,560 [51.97]	119,774 [44.55]
**Sex**			
Male	134,438 [51.31]	3,447 [50.32]	137,885 [51.28]
Female	127,576 [48.69]	3,403 [49.68]	130,979 [48.72]
**Parental Income[c]**			
Low	11,512 [4.39]	1,656 [42.36]	13,168 [4.95]
Lower-middle	98,268 [37.50]	1,927 [49.30]	100,195 [37.68]
Upper-middle	109,823 [41.91]	256 [6.55]	110,079 [41.40]
High	42,411 [16.19]	70 [1.79]	42,481 [15.97]
**Parental Education[d]**			
Primary	15,385 [5.87]	82 [4.47]	15,467 [5.86]
Secondary	75,377 [28.77]	264 [14.39]	75,641 [28.67]
Tertiary	171,252 [65.36]	1,489 [81.14]	172,741 [65.47]
**6th grade children**			
**Weight Group[a]**			
Underweight	3,481 [2.25]	92 [2.54]	3,573 [2.26]
Normal weight	118,794 [76.87]	2,457 [67.74]	121,251 [76.66]
Overweight	25,214 [16.31]	749 [20.65]	25,963 [16.41]
Obesity	7,058 [4.57]	329 [9.07]	7,387 [4.67]
**COVID-19 pandemic period[b]**			
Before	50,876 [32.92]	925 [25.27]	51,801 [32.74]
During	30,476 [19.72]	542 [14.81]	31,018 [19.61]
After	73,195 [47.36]	2,193 [59.92]	75,388 [47.65]
**Sex**			
Male	79,133 [51.20]	1,834 [50.11]	80,967 [51.18]
Female	75,414 [48.80]	1,826 [49.89]	77,240 [48.82]
**Parental Income[c]**			
Low	5,147 [3.33]	860 [41.01]	6,007 [3.83]
Lower-middle	47,582 [30.79]	1,083 [51.65]	48,665 [31.07]
Upper-middle	67,049 [43.38]	117 [5.58]	67,166 [42.88]
High	34,769 [22.50]	37 [1.76]	34,806 [22.22]
**Parental Education[d]**			
Primary	9,007 [5.83]	56 [5.92]	9,063 [5.83]
Secondary	50,764 [32.85]	155 [16.38]	50,919 [32.75]
Tertiary	94,776 [61.33]	735 [77.70]	95,511 [61.42]

[a]Age- and sex-adjusted Body Mass Index (iso-BMI) weight categories from IOTF thresholds; underweight (iso-BMI ≤ 18.5 kg/m^2), normal weight (iso-BMI 18.5-24.9 kg/m^2), overweight (iso-BMI 25-30 kg/m^2), obesity (iso-BMI ≥ 30 kg/m^2). [b]COVID-19 time periods; Before (11/3/19-10/3/20), During (1/9/20-30/6/ 21), After (1/8/22-31/1/24). [c]Parental equivalized disposable household income categorized by Danish year-specific quartiles, low (<25%), lower-middle (25-49.9 %), upper-middle (50-75%), high (>75%). [d]International Standard Classification of Education (ISCED) level of parents' highest completed education.

© 2025 Jensen FK et al. *JAMA Network Open*.
