# Supplementary material for: Body Mass Index in Children Before, During, and After the COVID-19 Pandemic
Source: JAMA Netw Open. 2025 Jul 9;8(7):e2519528. doi: 10.1001/jamanetworkopen.2025.19528 (PMC12242698; doi:10.1001/jamanetworkopen.2025.19528)
Supplement: Supplement 2. — Data Sharing Statement [file jamanetwopen-e2519528-s002.pdf]

Data Sharing Statement

Jensen. Body Mass Index in Children Before, During, and After the COVID-19 Pandemic. *JAMA Netw Open*. Published July 09, 2025. doi:10.1001/jamanetworkopen.2025.19528

Data
Data available: No

Additional Information
Explanation for why data not available: Data are available as presented in the paper. However, due to Danish legislation, our approvals for using these Danish data sources do not permit us to share or distribute them to third parties. Researchers interested in accessing the data can apply through the Research Service at the Danish Health Data Authority. Access to data from the Danish Health Data Authority requires approval from the Danish Data Protection Agency. The authors do not have special access privileges to these data.
